# Macroscale Superlubricity on Nanoscale Graphene Moiré Structure‐Assembled Surface via Counterface Hydrogen Modulation

**DOI:** 10.1002/advs.202309701

**Published:** 2024-03-14

**Authors:** Yongfu Wang, Xing Yang, Huiting Liang, Jun Zhao, Junyan Zhang

**Affiliations:** ^1^ State Key Laboratory of Solid Lubrication Lanzhou Institute of Chemical Physics Chinese Academy of Science Lanzhou 730000 China; ^2^ Key Laboratory of Science and Technology on Wear and Protection of Materials Lanzhou Institute of Chemical Physics Chinese Academy of Sciences Lanzhou 730000 China; ^3^ Division of Machine Elements Department of Engineering Sciences and Mathematics Luleå University of Technology Luleå SE‐97187 Sweden; ^4^ Center of Materials Science and Optoelectronics Engineering University of Chinese Academy of Sciences Beijing 100049 China

**Keywords:** counterface hydrogen modulation, graphene moiré structures, strain engineering, superlubricity, van der Waals

## Abstract

Interlayer incommensurateness slippage is an excellent pathway to realize superlubricity of van der Waals materials; however, it is instable and heavily depends on twisted angle and super‐smooth substrate which pose great challenges for the practical application of superlubricity. Here, macroscale superlubricity (0.001) is reported on countless nanoscale graphene moiré structure (GMS)‐assembled surface via counterface hydrogen (H) modulation. The GMS‐assembled surface is formed on grinding balls via sphere‐triggered strain engineering. By the H modulation of counterface diamond‐like carbon (25 at.% H), the wear of GMS‐assembled surface is significantly reduced and a steadily superlubric sliding interface between them is achieved, based on assembly face charge depletion and H‐induced assembly edge weakening. Furthermore, the superlubricity between GMS‐assembled and DLC25 surfaces holds true in wide ranges of normal load (7–11 N), sliding velocity (0.5–27 cm ^−1^s), contact area (0.4×10^4^–3.7×10^4^ µm^2^), and contact pressure (0.19–1.82 GPa). Atomistic simulations confirm the preferential formation of GMS on a sphere, and demonstrate the superlubricity on GMS‐assembled surface via counterface H modulation. The results provide an efficient tribo‐pairing strategy to achieve robust superlubricity, which is of significance for the engineering application of superlubricity.

## Graphical abstract

By sphere‐triggered strain engineering, the graphene moiré structure (GMS)‐assembled surfaces are formed. The superlubricity between GMS‐assembled and DLC25 surfaces in wide ranges of normal load (7–11 N), sliding velocity (0.5–27 cm s^−1^), contact area (0.4×10^4^–3.7×10^4^ µm^2^), and contact pressure (0.19–1.82 GPa) can be achieved based on assembly face charge depletion and H‐induced assembly edge weakening.

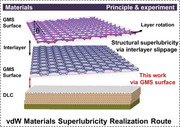

## 1 Introduction

Friction causes considerably energy loss and mechanical component failure in the worldwide every year (costing approximately 119 EJ).^[^ ^1^, ^2^, ^3^ ^]^ Moiré structural van der Waals (vdW) materials can provide a near‐frictionless state (termed superlubricity) due to their weak interlayer vdW interactions in the incommensurate state.^[^ ^4^, ^5^ ^]^ Achievement of superlubricity has been proved in homogeneous (graphite/graphite,^[^ ^6^, ^7^, ^8^ ^]^ graphene/graphene,^[^ ^9^, ^10^ ^]^ MoS_2_/MoS_2_^[^ ^11^, ^12^ ^]^) and heterogeneous (graphite/MoS_2_,^[^ ^13^, ^14^ ^]^ graphite/hexagonal boron nitride,^[^ ^15^, ^16^, ^17^ ^]^ gold/graphite,^[^ ^18^, ^19^ ^]^ Sb/graphite,^[^ ^20^ ^]^ Sb/MoS_2_^[^ ^21^ ^]^) contacts. In order to maintain interlayer flatness slippage, the involved vdW materials are either pristine single‐crystals or ones pre‐prepared on atomically flat supporting substrates by wet transfer process or chemical vapor deposition.^[^ ^22^, ^23^, ^24^, ^25^ ^]^ However, the actually used substrates in engineering exhibit relatively high roughness (generally higher than 100 nm, corresponding to the highest class 0 in the VDI 3400‐surface standard^[^ ^26^ ^]^), which puckers and even ruptures the vdW layers and increases friction.^[^ ^24^, ^25^, ^27^ ^]^ On the other hand, the superlubricity in the homogeneous and heterogeneous contacts shows a twist‐angle dependence.^[^ ^6^, ^9^, ^15^ ^]^ Layers undergo a spontaneous rotation to high‐friction commensurate state when sliding occurs, because the incommensurate contact is fundamentally an instable state.^[^ ^9^, ^28^, ^29^ ^]^ This instable state leads to the disappearance of superlubricity.^[^ ^6^, ^9^ ^]^ The dilemma makes the larger scale acquirement of incommensurateness via interlayer slippage being limited to few special structure vdW materials which have long periodicity and perfect atomic structures, such as strained bilayer graphene^[^ ^30^ ^]^ and double‐walled carbon nanotubes (CNT).^[^ ^31^ ^]^ Due to the above challenges, interlayer superlubricity can only be achieved under limited conditions such as super‐smooth substrate, ultralow applied load and extra‐small contact area etc.,^[^ ^28^, ^32^, ^33^ ^]^ which poses a major issue for engineering applications of superlubricity. This state of affairs inspires us to explore other pathway through which moiré structural vdW materials will enable to provide superlubricity.

The interlayer sliding within moiré structural vdW materials produces structure superlubricity.^[^ ^32^, ^34^, ^35^ ^]^ Alternatively, the stabilizing of moiré structures by strong interlayer chemical action avoids the interlayer sliding, which makes them an opportunity to act as a frictional surface. This provides a new way to achieve superlubricity via the surface of moiré structures but not their interlayer slippage. Thus, it is crucial to explore how to form moiré structures with interlayer chemical action and form a superlubric tribo‐pair with which kinds of sliding counterbody. In this context, a spherical substrate is more preferable to solve the first obstacle, since when 2D flat vdW planes adhere to a spherical surface, the planes are spontaneously strained to produce lattice mismatch and form moiré structures, unnecessary for additional human assistance as prerequisite. On the other hand, counterface H modulation is feasible to solve the latter obstacle, because the least sliding resistance needed for moiré structural vdW materials is acquired by no interfacial atomic bonding of H‐termination counterbody, such as hydrogenated diamond‐like carbon (DLC).^[^ ^36^, ^37^, ^38^ ^]^

**Figure 1:**
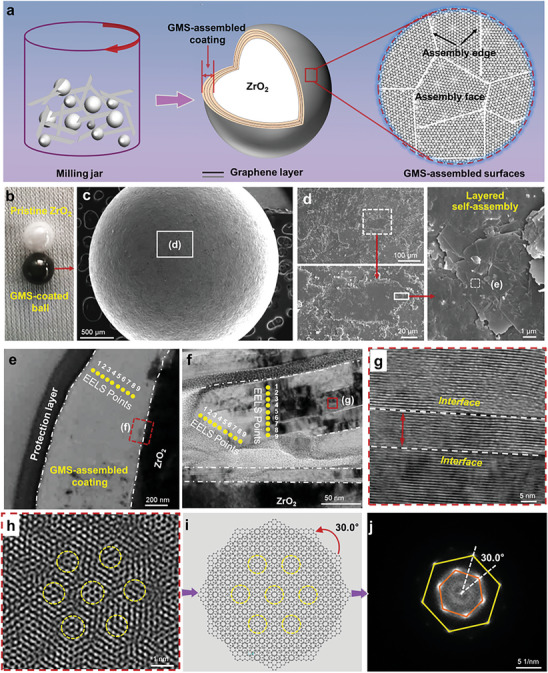
Establishment of GMS‐assembled coating. a) Preparation schematic diagram of GMS‐assembled coating. b) ZrO_2_ balls before and after ball‐milling. c,d) Scanning electron microscopy (SEM) images of GMS‐assembled coating. e–g) TEM cross‐section images of GMS‐assembled coating, (f) is marked in (e), (g) is marked in (f). h) Moiré patterns of GMS‐assembled coating cut from Figure S1 (Supporting Information). i) Schematic moiré patterns of (h). j) FFT of moiré patterns of (h).

Here, we demonstrate macroscale superlubricity on nanoscale graphene moiré structure (GMS)‐assembled surface via counterface H modulation. By the sphere‐triggered strain engineering under the co‐effect of edge sp^3^‐C formation, coating compaction and strong coating‐ball adhesion, countless nanoscale GMS are assembled into a whole coating on grinding balls, thereby forming GMS‐assembled surface. The ball‐supported GMS‐assembled surface has extremely low wear under the H modulation of counterface diamond‐like carbon (25 at.% H, named as DLC25), which produces a steadily superlubric sliding interface between them, based on assembly face charge depletion and H‐induced assembly edge weakening (**Figure** **1**a). Furthermore, the superlubricity between GMS‐assembled and DLC25 surfaces holds true in wide ranges of normal load (7–11 N), sliding velocity (0.5–27 cm ^−1^s), contact area (0.4×10^4^–3.7×10^4^ µm^2^) and contact pressure (0.19–1.82 GPa), among which an extremely low friction coefficient of 0.001 is recorded during 6×10^4^ cycles. Atomistic simulations confirm the preferential formation of GMS on a sphere, and demonstrate the superlubricity on GMS‐assembled surface via counterface H modulation. The superlubric ranges are much larger than those in previously reported works, which greatly helps to bridge superlubricity to real applications. This work provides a facile tribo‐pairing way to achieve macroscale superlubricity of vdW materials, and also develops an efficient strategy for the synthesis of moiré structural vdW materials.

## 2 Results and Discussion

### 2.1 Material Formation and Characterization

Less‐defective and few‐layer graphene flakes with the size of 500 nm are directly used to form GMS, through dry ball milling with different‐sized ZrO_2_ balls (3, 5, and 10 mm diameter, Figure 1a). These nanoscale GMS self‐assemble into a whole coating on top of the balls, resulting in a dramatic color change (the white ball before grinding is changed into a black one, Figure 1b–d). Figure 1e,f shows the transmission electron microscopy (TEM) cross‐section of GMS‐assembled coating. It is found that graphene flakes lie flatly on the ball surface, and they have obvious boundaries with each other. This unique stacking structure suggests the self‐orientated layer‐by‐layer assembly of graphene flakes along the ball surface.^[^ ^39^, ^40^ ^]^ The self‐orientated assembly of graphene flakes can effectively sprawl along the ZrO_2_ ball surface, thereby completely encapsulating the balls as displayed in Figure 1b,c.

A striking finding is the orientational misalignment between adjacent graphene sheets in the stacking structure (Figure 1h–j). The TEM image of the graphene flakes exfoliated from the ball‐supported GMS‐assembled coating reveals that graphene sheets can form moiré patterns (Figure 1h). The schematic illustration of simulation (Figure 1i) and the Fast Fourier transformation (FFT) image (Figure 1j) obtained from the 2025 nm^2^ area of TEM image (Figure S1, Supporting Information) prove that there is a mismatch angle of 30.0° between the sheets. The big stacking angle and large‐area moiré patterns suggest the presence of incommensurate contact between graphene sheets on grinding balls, and also confirm the GMS formation. The ball‐supported GMS‐assembled coating acts as a frictional surface and makes up a tribo‐pair with counterface DLC that is modulated by H, which is used to attain superlubricity.

### 2.2 GMS Formation Mechanism

**Figure 2:**
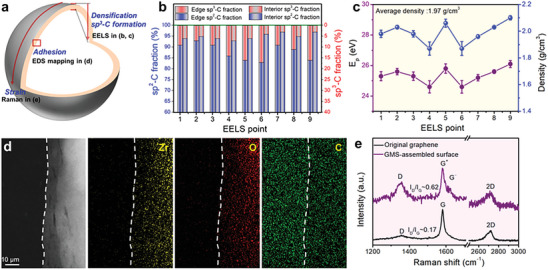
Characterization of GMS‐assembled coating. a) Analytical method illustration of GMS‐assembled coating. b) Evolution of edge and interior sp^3^‐C bonds obtained from EELS points in Figure 1f. c) Evolution of mass densities and bond fractions obtained from EELS points in Figure 1e. d) HADDF and TEM‐EDS mapping images of Zr, O and C elements. e) Typical Raman spectra of GMS‐assembled coating and original graphene.

Here, we will discuss the reasons for GMS formation on ZrO_2_ balls (**Figure** **2**a). First, it is found that the GMS‐assembled coating contains a certain amount of sp^3^‐bonded carbon (sp^3^‐C), and the fraction of sp^3^‐C at the graphene edge is higher than that at the graphene interior (Figure 2b). This is supported by the edge and interior sp^3^‐C fraction (7% −17% vs 3% −7%) calculated from core‐less electron energy loss spectra (EELS, Figure 2b; Figure S2a, Supporting Information) by a multiple‐functional fitting approach.^[^ ^41^, ^42^, ^43^ ^]^ The low content of interior sp^3^‐C has no effect on the fundamental structures of graphene flakes, that is, it maintains the 2D material integrity. This can be verified by the observation of the near‐intact graphene basal planes in the TEM image (Figure 1g). The edge sp^3^‐C provides chemical linkages among adjacent graphene flakes, thereby resulting in the assembling of countless nanoscale GMS into a whole coating. On the other hand, the density of GMS‐assembled coating is estimated by the equation related with the plasmon peak *E*_p_ in zero‐loss EELS.^[^ ^38^ ^]^ The coating density is averaged to be 1.97 g cm^−3^ over five measured points (Figure 2c; Figure S2b, Supporting Information), which is close to that of highly oriented pyrolytic graphite (2.16 g cm^−3^).^[^ ^43^ ^]^ Together the edge sp^3^‐C formation and coating compaction above integrate many individual graphene flakes into a whole and densifying coating. Interestingly, the high‐angle annular dark‐field (HADDF) and TEM energy dispersive spectroscopy (TEM‐EDS) mapping images of C and Zr (or O) elements show that many carbon atoms permeate into ZrO_2_ substrates (Figure 2d). This permeating effect leads to the strong coating‐ball adhesion that triggers the whole coating bend along the spherical surface and makes the embedded graphene sheets being in a strained state. The strained state is further evidenced by the more obviously splitting of G peak^[^ ^44^, ^45^ ^]^ in the Raman spectra as the measured surface gets closer to the underlying ball (Figure 2e; Figure S3, Supporting Information). The more splitting of G peak close to the underlying ball indicates higher levels of strain from outermost surface to near‐ball surface, where its lattice constant is somewhat different between up–down graphene sheets being in a different strained state. The different levels of strain have been proved to induce a lattice constant mismatch between adjacent graphene sheets,^[^ ^30^, ^46^ ^]^ resulting in the occurrence of incommensurable state. By the sphere‐triggered strain engineering under the co‐effect of edge sp^3^‐C formation, coating compaction and strong coating‐ball adhesion, countless nanoscale GMS are assembled into a whole coating on the grinding balls, thereby forming GMS‐assembled surface.

It should be noted that graphene flakes in the GMS‐assembled coating are of various shapes, sizes and orientations, which affects strain space distribution and leads to various incommensurable contact states. As expected, the TEM images of graphene flakes extracted from the other coated ball positions show the various contact states such as 0° and 30.0° in Figure S4 (Supporting Information) and 8.5°, 26.0° and 34.8° in Figure S5 (Supporting Information). The various incommensurable contact states have interesting ramifications for the friction processes of GMS‐assembled coating on the H‐modulated DLC surface, which is further discussed by a combination of experiment and theoretical simulation methods.

### 2.3 Counterface‐H‐Induced Superlubricity

**Figure 3:**
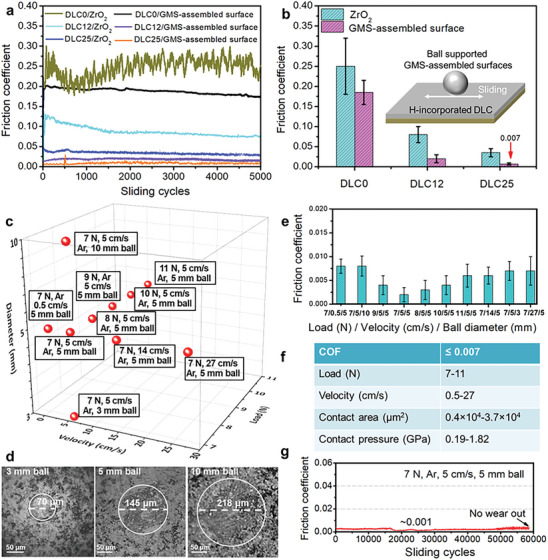
Friction behaviors. a) Friction coefficients under the tribo‐pairing of DLC0, DLC12, DLC25 at 7 N, 5 cm ^−1^s, and 3 mm ball. b) Average friction coefficients of (a). c) Superlubric ranges of GMS‐assembled coating sliding against DLC25. d) Superlubric wear scars of ball‐supported (3, 5, and 10 mm) GMS‐assembled coating sliding against DLC25 at 7 N and 5 cm ^−1^s. e) Average friction coefficients obtained from (c). f) Friction coefficient, normal load, sliding velocity, contact area, and contact pressure ranges obtained from (e). The steady‐state apparent average contact pressure is calculated based on (d). g) Friction coefficients for GMS‐assembled coating sliding against DLC25 at 7 N, 5 cm ^−1^ s, and 5 mm ball.

Having determined the GMS formation on ZrO_2_ balls, we investigated the friction behaviors of GMS‐assembled coating under the H modulation of counterface DLC in dry Ar gas. The series of DLC were synthetized by a magnetron sputtering technique using the mixed Ar/H_2_ gases with the flow ratios of 1:0, 1:2, and 1:6 (Figure S6, Supporting Information). The H content of the 1:2 and 1:6 samples are about 12 and 25 at.%, respectively,^[^ ^47^ ^]^ which is far lower than the known H content threshold value of achieving DLC's superlubricity (H>40 at.%).^[^ ^36^, ^37^, ^38^ ^]^ The 1:0, 1:2 and 1:6 DLC samples are named as DLC0, DLC12, DLC25, respectively. The GMS‐assembled surface (Ф3 mm) was driven to slide against these DLC in a reciprocating mode at the sliding velocity of 5 cm ^−1^s and the normal applied load of 7 N. The average friction coefficients of GMS‐assembled coating on the DLC0, DLC12, DLC25 samples are 0.186, 0.031, and 0.007, respectively, indicating that superlubricity is easier to be realized under the more H modulation of counterface DLC (**Figure** **3**a,b). Whereas the friction coefficients of pure ZrO_2_ balls on the DLC0, DLC12, DLC25 are 0.247, 0.083, and 0.033, respectively (Figure 3a,b), verifying the key role of counterface H modulation in the superlubricity achievement of GMS‐assembled coating. The counterface H modulation provides least sliding resistance needed for superlubricity achievement under the interfacial chemical weakening. Note that the excessive hydrogen incorporation of DLC leads to the superlubricity failure (Figure S7, Supporting Information). On the other hand, the superlubricity regime of GMS‐assembled coating sliding against DLC25 occurs over a wide range (Figure 3c–g; Figure S8, Supporting Information), that is, the super‐low friction coefficients less than 0.007 can be obtained when the external load varies from 7 to 11 N, the sliding velocity changes from 0.5 to 27 cm ^−1^s, the contact area increases from 0.4×10^4^ µm^2^ (3 mm ball) to 3.7×10^4^ µm^2^ (10 mm ball) and the corresponding steady‐state apparent average contact pressure *P* changes from 0.19 to 1.82 GPa. Especially at the conditions of 7 N, 5 cm ^−1^s, and 5 mm ball, an extremely low friction coefficient *µ* of 0.001 with very low amplitude of variation (±0.0005) is recorded and approaches for 6×10^4^ cycles. The corresponding interfacial shear strength *S*_0_ of the extremely low friction is 0.43 MPa, according to the equation *µ* = *S*_0_/*P*,^[^ ^48^ ^]^ where the *P* in the steady state is 0.43 GPa (when the diameter of wear scar is 145 µm). The shear strength is far higher than the interlayer shear strength of millimeter scale graphene in the superlubricity state (0.07 MPa),^[^ ^15^ ^]^ indicating that the superlubricity of GMS‐assembled coating versus DLC25 does not rely on the interlayer slippage within graphene moiré structures but their interfacial interaction with DLC25. The failure of interlayer slippage within graphene moiré structures should due to the presence of sp^3^‐C at the graphene interior and edge (Figure 2b).

### 2.4 Counterface‐H‐Induced Ultra‐Low Wear

**Figure 4:**
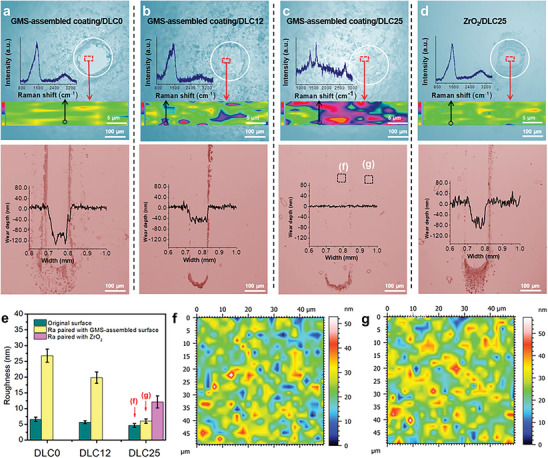
Wear behaviors. a–d) Laser‐interference surface images, Raman mapping images and Raman spectra of the wear scars and tracks of GMS‐assembled coating/DLC0, GMS‐assembled coating/DLC12, GMS‐assembled coating/DLC25 and ZrO_2_/DLC25 tribo‐pairs. e) Average roughness. f,g) 2D morphologies of wear track inside and outside marked in (c).

To confirm the interfacial interaction between GMS‐assembled coating and DLC, the wear features on their wear tracks and scars are analyzed. As shown in **Figure** **4**a–d, under more H‐incorporated DLC, the GMS‐assembled coating is more able to reside on the ZrO_2_ balls. This is confirmed by the gradually decreased material transfer toward the DLC surface (Figure 4a–d). The Raman spectra obtained from the wear tracks from DLC0 to DLC25 further show the stable existence of GMS‐assembled coating on the ball surface under more H‐incorporation (Figure 4a–d). The obviously reduced material transfer is attributed to the survival of DLC25's smooth surface, which is supported by the wear depth and surface roughness changes (Figure 4a–g). The wear depth decreases from ≈120 nm of DLC0 to near‐zero nm of DLC25, and the counterface‐H‐induced ultra‐low wear is revealed by TEM cross‐section images obtained from GMS‐assembled and DLC25 surfaces (Figure S9, Supporting Information). The roughness of DLC25 wear track is 6.0 nm, which is slightly higher than its initial roughness (4.6 nm, Figure 4f,g; Figure S10, Supporting Information). The survival of the DLC25's smooth surface achieves a weak cross‐interfacial mechanical interlocking, which avoids the local puckering of graphene flakes in GMS‐assembled coating and reduces the flakes’ mechanical exfoliation,^[^ ^49^, ^50^ ^]^ thereby making the coating stably existing on the ball surface. This allows GMS‐assembled coating to act as a frictional surface and combine with DLC25 surface to form a robust sliding interface. On the other hand, the high passivation of C─H bonds to DLC25 surface remarkablely reduces the π bond content of DLC surface and weakens cross‐interface π–π^*^ interaction between DLC25 and GMS‐assembled surfaces,^[^ ^51^, ^52^, ^53^, ^54^ ^]^ which leads to a weak cross‐interface chemical adhension. The weaker cross‐interface chemical adhension obtained by the pairing of H‐richer DLC attains a lower friction coefficient (Figure 3b). By the weak mechanical interlocking and cross‐interface chemical adhension under the H modulation of counterface DLC, the extremely low wear of GMS‐assembled coating is achieved, which produces a steady sliding interface and promotes the achievement of macroscale superlubricity between them.

In addition, the extremely low material transfer of GMS‐assembled coating toward DLC25 surface (i.e., wear) is related with the local recovery of GMS’ interlayer slippage. This is verified by the Raman spectra of wear debris at the GMS‐assembled coating/DLC25 sliding track end which show the slightly decreased D/G intensity ratio from original 0.62–0.50 (Figure S11, Supporting Information). The decreased D/G intensity ratio means the reduction of sp^3^‐bonded carbon fraction, implying the local recovery of interlayer slippage.^[^ ^55^, ^56^, ^57^ ^]^ The local recovery of interlayer slippage would enhance the wear of GMS‐assembled coating and promote cross‐interface material transfer, reducing the robustness of superlubric sliding and even leading to the superlubric failure, such as in the two sides of DLC0 wear track (Figure 4a).

All the above results demonstrate that the GMS‐assembled coating formed on the grinding balls under sphere‐triggered strain engineering achieves robust macroscale superlubricity by counterface H modulation.

### 2.5 Theoretical Simulations

**Figure 5:**
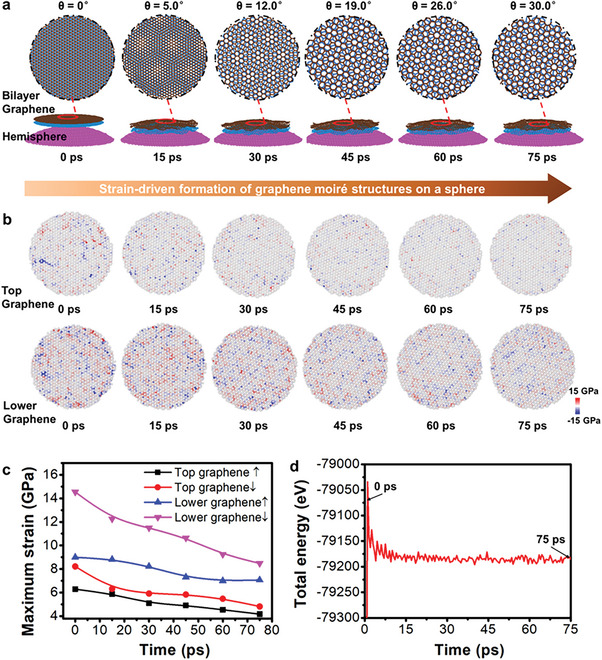
MD simulations of GMS formation. a) Atomic snapshots (0 and 75 ps) of GMS formation on a hemisphere. b) Strain distribution maps at each snapshots of top and lower graphene. The blue and red represent upward and downward strain. c) Maximum strain results. d) Total energy changes.

To elucidate the achievement mechanism of superlubricity via GMS‐assembled surface and counterface H modulation, the GMS formation process on a sphere as well as the friction behaviors of GMS‐assembled coating on DLC surface are investigated via theoretical simulation method. First, we have investigated the GMS formation mechanism on a rigid hemisphere by molecular dynamics (MD) simulations. It is indicated that the commensurable bilayer graphene (CBG) readily conforms to the hemisphere surface, reflecting the bottom sphere topography and triggering commensurate‐incommensurate transition (the mismatch angle changes from θ = 0° to θ = 30.0°) in the bending process that results in the GMS formation (**Figure** **5**a; Movie S1, Supporting Information). Corresponding force analysis of the bending process of bilayer graphene is used to identify the strain level from top graphene to lower one. The downward strain is higher than the upward strain at each snapshots, which bends individual layers along the bottom sphere and leads to the strain reduction (Figure 5b,c; Movie S1, Supporting Information). Nevertheless, the lower graphene is under higher strain than the top, and its lattice constant is somewhat different from the top under higher downward strain. The different levels of strain from top graphene to lower one induces a mismatch between their lattice constants,^[^ ^30^, ^46^, ^58^ ^]^ which leads to an incommensurate state. Besides, analysis of the system energy supports the energetically favorable formation of GMS on a sphere, where the system energy decreases from −79 036 eV (at 0 ps) to −79 186 eV (at 75 ps) (Figure 5d). The results clearly support the strain‐driven formation of graphene moiré structures on grinding balls, where sphere‐triggered strain engineering enables graphene sheets embedded in the coating to produce incommensurate contact and form the structures.

**Figure 6:**
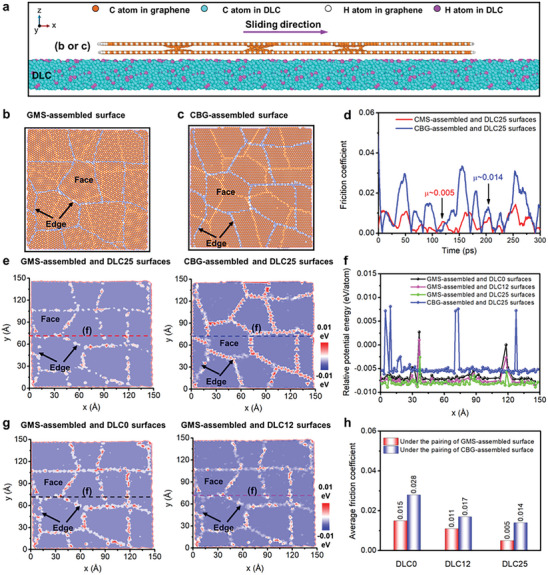
MD simulation between GMS‐assembled and DLC surfaces. a) Friction models of eight GMS‐assembled and CBG‐assembled flake on DLC. b,c) Structure models. d) Friction results. e) Relative potential energy images between GMS‐assembled and DLC25 surfaces, and between CBG‐assembled and DLC25 surfaces. f) The energy curves as marked in (e,g). g) Relative potential energy images between GMS‐assembled and DLC0 surfaces, or GMS‐assembled and DLC12 surfaces, respectively. h) Friction results on DLC surfaces under the tribo‐pairing of GMS‐assembled and CBG‐assembled surfaces.

Second, the friction behaviors of GMS‐assembled coating are investigated by the shear sliding along the x direction of eight nano‐sized GMS‐assembled flake on DLC25 surface (**Figure** **6**a,b; Movie S2, Supporting Information). The nano‐sized GMS have different moiré patterns, which is consistent with various incommensurable contact states in the ball‐supported GMS‐assembled coating (Figures S4 and S5, Supporting Information). The MD simulation of the shear sliding at the sliding velocity of 20 m ^−1^s shows robust superlubric behavior (0.005, Figure 6d). In comparison, the eight nano‐sized CBG‐assembled flake exhibits a friction coefficient of 0.014 under the same sliding conditions (Figure 6c,d). This is supported by corresponding relative potential energy analysis of GMS‐assembled and CBG‐assembled surfaces under the pairing of DLC25 surface, where the relative potential energies on individual GMS face and edge have more obvious reduction than those of individual CBG (energy value on faces: −0.0083 eV per atom vs −0.0052 eV per atom; energy value on edges: −0.0025 eV per atom vs 0.0087 eV per atom, Figure 6f). On the other hand, the simulations of the shear sliding of eight nano‐sized GMS‐assembled flakes on DLC0, DLC12 and DLC25 surfaces shows that the friction coefficients of GMS‐assembled flake on DLC0, DLC12 and DLC25 surfaces are 0.015, 0.011 and 0.005, respectively (Figure 6h). In sharp contrast, the friction coefficients of CBG‐assembled flake on the surfaces are 0.028, 0.017 and 0.014, respectively. The high friction is also observed in the CBG‐assembled flake having different face orientations (Figure S12, Supporting Information). These results verify the key role of counterface H modulation in the superlubricity achievement of GMS‐assembled coating. Corresponding relative potential energy analysis shows that the GMS‐assembled surface has lower energy under more H‐modulation of counterface DLC, where the energy value on faces decreases from −0.0063 eV per atom, −0.0072 eV per atom to −0.0083 eV per atom, and the energy value on edges decreases from 0.0028 eV per atom, 0.0015 eV per atom to −0.0025 eV per atom (Figure 6e–g). Analysis of the relative potential energy supports the counterface‐H‐induced weakening of assembly edges and faces which leads to a weak cross‐interface chemical adhension. The weak cross‐interface chemical adhension bestows the GMS‐assembled surface extremely low wear, and also produces a steadily superlubric sliding interface between GMS‐assembled and DLC25 surfaces.

**Figure 7:**
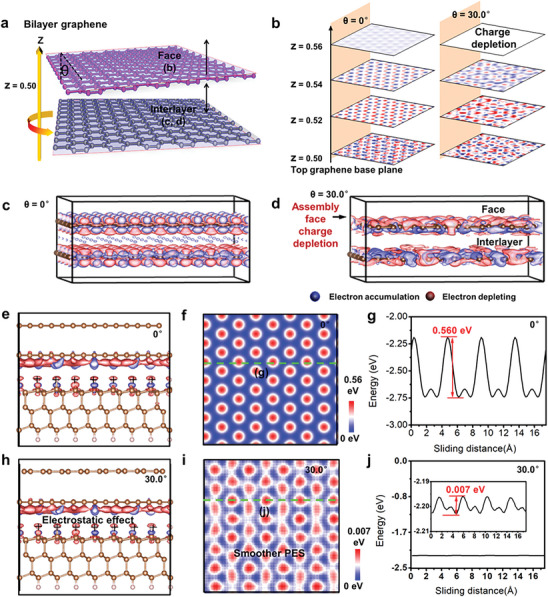
DFT calculations of interface between GMS face and DLC25. a) Schematic illustration of the surface and interlayer of bilayer graphene. b) 2D electron density difference maps for the GMS and CBG surfaces at different perpendicular heights as marked in (a). The twisted angles between bilayer graphene of GMS and CBG are 0° and 30.0°, respectively. c,d) 3D electron density difference maps (0.0005 e Å^−3^) of GMS and CBG showing an assembly face charge depletion behavior. e–g) Electron density differences (e), and PES corrugation (f) and curves (g) of CBG/H‐diamond interface. h–j) Electron density differences (h), and PES corrugation (i) and curves (j) of GMS/H‐diamond interface. The blue and red in the (c–e,h) represent electron accumulation and depletion.

Following, we noted that individual GMS face has the relative potential energy of −0.0083 eV per atom which is lower than that of individual CBG face (−0.0052 eV per atom) under the pairing of DLC25 surface, indicating the change of their in‐plane interactions. The in‐plane interactions are investigated by the density functional theory (DFT) calculations of bilayer graphene after layer rotating from θ = 0° to θ = 30.0° (**Figure** **7**a; Figures S13 and S14, Supporting Information). In Figure 7b–d, the commensurate‐incommensurate transition induces an obvious charge depletion behavior on both the surface and interlayer, that is, the moiré structure triggers the charge depletion of assembly face. The assembly face charge depletion allows the positively charged proton in the nucleus of θ = 30.0° system to be closer to the surface (Figure 7d), bestowing GMS a positively charged face. The positively charged face gives rise to electrostatic repulsion with the hydrogenated diamond‐like carbon surfaces that are known to be charged positively,^[^ ^36^, ^37^ ^]^ which leads to the lower face energy on GMS‐assembled surface under the more counterface H modulation (Figure 6f). The effect of moiré structure‐triggered charge depletion on the friction is further investigated by the DFT calculations of single GMS (θ = 30.0°) and CBG (θ = 0°) flakes on hydrogenated diamond (H‐diamond, 25 at.% H). It can be seen that, the GMS and H‐diamond interface has smaller electron density, weaker interfacial energy (−2.20 eV vs −2.74 eV) and more smooth sliding potential energy surface (PES) than the CBG and H‐diamond interface (Figure 7e–j; Figures S13 and S14, Supporting Information). The average energy difference between the peaks and valleys of GMS in the PES images is 0.007 eV (Figure 7j), approximately 1/80 of CBG (0.560 eV, Figure 7g), enabling more effortless superlubric sliding with lower sliding energy barrier.^[^ ^17^, ^59^ ^]^ The results confirm that the assembly face charge depletion in incommensurate state helps to the weakening of cross‐interfacial chemical adhesion between GMS‐assembled and DLC25 surfaces and promotes the achievement of macroscale superlubricity.

Subsequently, because the parallel stacking of variously shaped, sized and thickened graphene flakes along the ZrO_2_ balls leads to the surface roughening of GMS‐assembled coating, we investigated the effect of the roughness on their superlubricity behaviors. The MD simulation was performed under the same conditions with the non‐stacking system in Figure 6a, and the surface roughness was controlled by the number of GMS flakes parallelly stacked on the GMS‐assembled surface. It is indicated that the friction coefficients of one, two and four GMS flakes on DLC25 surface are 0.006, 0.007, and 0.009, respectively (Figure S15, Supporting Information), Whereas the friction coefficients of one, two and four CBG flakes are 0.015, 0.017, and 0.020, respectively (Figure S16, Supporting Information). The individual GMS flake acts as a single asperity contact and divides surface contact into point contact; however, because of new edge formation in the discontinuous stacking process, the reduced contact area only produces superlubricity but does not generate a friction coefficient which is lower than non‐stacking system (0.005). The results support the situation that the macroscopic contact of GMS‐assembled coating formed by the parallel stacking of variously shaped, sized and thickened graphene flakes along the ZrO_2_ balls resembles a multiple asperity contact, during which a large number of GMS asperities bridge nanotribology and macroscale superlubricity. Once the wear of GMS‐assembled coating is significantly reduced under counterface H modulation, a steadily superlubricious state can be achieved, based on assembly face charge depletion and H‐induced assembly edge weakening.

## 3 Conclusion

In summary, we deposited GMS‐assembled coatings on the ZrO_2_ balls using the ball‐milling process and tested its friction properties against the hydrogenated DLC surface for a range of sliding velocities and applied contact load conditions. The results indicate that once the graphitic layers on the ball side rotate to arrange in Moiré patterns, the friction drops below the superlubricity threshold. Wear studies demonstrate that the wear of GMS‐assembled surface is significantly reduced under the H modulation of counterface DLC25. This significantly reduced wear produces a steady sliding interface between GMS‐assembled and DLC25 surface, achieving robust superlubricity based on assembly face charge depletion and H‐induced assembly edge weakening. Atomistic simulations support the experimental findings and correlate the strain‐guided rotation of graphitic layers with the changes in the coefficient of friction. This work establishes an efficient route to synthesize moiré structural vdW materials, and also provides a feasible tribo‐pairing strategy to attain macroscale superlubricity.

## 4 Experimental Section

### Material Preparation

DLC coatings including DLC0, DLC12 and DLC25 (with a thickness of 500 nm)^[^ ^47^ ^]^ were prepared on Si substrates by an unbalanced magnetron sputtering system using the mixed gas of Ar and H_2_ with the flow ratio of 1:0, 1:2, 1:6, 1:8, and 1:10. The preparation parameters of DLC included the substrate bias voltage of −700 V (pulsed frequency of 60 kHz and duty cycle of 0.6) and the carbon target power of 0.50 kW. Prior to the deposition, the deposition chamber was pumped down to 2×10^−3^ Pa followed by the introducing mixed gas. The deposition temperature controlled by plasma bombardment did not exceed 120 °C.

Graphene nanoflakes with the average length of 500 nm were purchased from Jiangsu XF NANO Materials Tech Co., Ltd. Most of the flakes had less than 10 nm thick and the purity was 99.9%. A certain amount of graphene (1 g) and ZrO_2_ balls (150 g, Ra 50 nm, ball diameter: 3, 5, and 10mm) were put into a milling jar, and then milled by the planetary ball milling machine (MITR QM‐QX‐2L, China) for 48 h with a rotation speed of 300 rpm. After ball‐milling, the ZrO_2_ ball‐supported samples were washed for at least three times using anhydrous ethanol and dried in a vacuum chamber (<0.1 Pa) at 60 °C for 3 h.

### Friction Experiments

All frictional tests were performed in a linear reciprocate mode by CSM (TRB^3^, Anton Paar, Switzerland) pin‐on‐disc tribometer. The GMS‐assembled coating was driven to slide on DLC in dry Ar atmosphere at room temperature. The chamber was introduced by dry Ar gas to produce dry environment with the relative humidity less than 5%. The sliding velocity changed from 0.5 to 27 cm ^−1^s, the normal load adjusted from 7 to 11 N, and the ball diameter varied from 3 to 10 mm, zero calibration of the machine was carried out automatically before each test. Each frictional test was repeated for 3 times to ensure the degree of precision.

### Characterization of Contact Regions

The structure and chemical composition of GMS‐assembled coating were detected by Raman spectrometer (Jobin‐Yvon HR‐800), scanning electron microscopy (SEM, JSM‐5601LV) and Transmission Electron Microscope (TEM, FEI Techanai G^2^). To avoid unintentional damage of the samples, the laser intensity in Raman tests was strictly controlled below 0.5 MWm^−2^, (because carbon materials did not suffer damage in this range^[^ ^43^, ^47^ ^]^). The Raman mapping images obtained from 8 µm×40 µm region were measured with an 1 µm step size in the y direction, a 3 µm step size in the x direction and an acquisition time of 10 s. The morphologies of ball‐milled balls, wear tracks and scars were analyzed using 3D white light interferometer (KLA‐Tencor, MicroXAM‐800) and optical microscope (Olympus‐BX35).

The lamellar specimens picked up from the GMS and wear track were prepared by a dual‐beam SEM/FIB system (Helios Nanolab 600) for TEM observation. Before FIB‐cutting, the GMS and wear track were coated by a Pt protective layer in an ion sputtered system. The lamellar specimens were cut under the operational voltage and current of 30 KV and 2.8 nA, further reduced to 100 nm (16 kV and 45 pA), and finally thinned to 20–50 nm (5 kV and 16 pA). The ultrathin thickness of the lamellar specimens over large areas helped to subsequent atomic‐resolution EELS and HAADF characterizations. The EELS and HAADF of GMS were measured under an energy resolution of 0.7 eV by using a spherical aberration corrected TEM (JEOL JEM‐ARM200F). In order to avoid structural damage, the accelerating voltage was controlled at 200 kV and the acquisition time was optimized to the range (0.05–0.10 s) in the EELS experiments.^[^ ^38^, ^60^ ^]^

### Theoretical Simulation

The formation mechanism of GMS on a hemisphere, as well as the friction process of GMS‐assembled coating on DLC surfaces (DLC0, DLC12 and DLC25) were studied by MD simulations employing adaptive interatomic reactive empirical bond‐order (AIREBO)^[^ ^61^ ^]^ and Lennard–Jones (L–J) potentials. In the simulation of GMS formation, we employed a circular bilayer graphene (40 Å of radius) on a rigid hemisphere (Text S1, Supporting Information). For the friction simulations of GMS‐assembled coatings, we constructed a GMS‐assembled flake (18 000 atoms) sliding on a DLC substrate (100 000 atoms), in which H atoms with the concentrations of 0%, 12% and 25% were randomly intercalated into DLC (Text S2, Supporting Information). All MD simulations were performed under NVT ensemble and 300 K with a time step of 1.0 fs in LAMMPS software. In order to reveal the superlubric nature of GMS‐assembled coating on the DLC surfaces, we employed standard DFT calculations in VASP software^[^ ^62^, ^63^ ^]^ to analyze the surface and interlayer properties (electron density, electron density difference, and PES) of bilayer graphene (224 atoms) after layer rotating from θ = 0° to θ = 30.0°, and estimated the interfacial energy changes of bilayer graphene and H‐diamond (406 atoms) (Text S3, Supporting Information). The generalized gradient approximation (GGA) with Perdew‐Burke–Ernzerhof (PBE) functional^[^ ^64^ ^]^ was used to describe the exchange‐correlation contributions. To reach structural optimizations, electron density distribution calculations and PES scan, the energy cutoff 400 eV for the plane‐wave basis was selected, and the energy and force thresholds were set to 10^−4^ eV and 0.05 eV Å^−1^, respectively. Such a combination of theoretical methods revealed the GMS formation mechanism, and the superlubric nature of GMS‐assembled surfaces under counterface H modulation.

## Conflict of Interest

The authors declare no conflict of interest.

## Supporting information

Supporting Information

Supplemental Movie 1

Supplemental Movie 2

## Data Availability

The data that support the findings of this study are available from the corresponding author upon reasonable request.
