# Supplementary material for: Macroscale Superlubricity on Nanoscale Graphene Moiré Structure‐Assembled Surface via Counterface Hydrogen Modulation
Source: Adv Sci (Weinh). 2024 Mar 14;11(19):2309701. doi: 10.1002/advs.202309701 (PMC11109616; doi:10.1002/advs.202309701)
Supplement: Supplementary file 1 — Supporting Information [file ADVS-11-2309701-s001.pdf]

ADVANCED SCIENCE
Open Access

Supporting Information

for *Adv. Sci.*, DOI 10.1002/advs.202309701

Macroscale Superlubricity on Nanoscale Graphene Moiré Structure-Assembled Surface via Counterface Hydrogen Modulation

Yongfu Wang, Xing Yang, Huiting Liang, Jun Zhao and Junyan Zhang**

Supporting Information

Macroscale superlubricity on nanoscale graphene moiré structure-assembled surfaces via counterface hydrogen modulation

Yongfu Wang, Xing Yang, Huiting Liang, Jun Zhao, Junyan Zhang**

Y. Wang, X. Yang, Huiting Liang, J. Zhang
State Key Laboratory of Solid Lubrication
Lanzhou Institute of Chemical Physics
Chinese Academy of Science
Lanzhou 730000, China
E-mail: yongfuwang@licp.cas.cn; zhangjunyan@licp.cas.cn

J. Zhao
Division of Machine Elements
Department of Engineering Sciences and Mathematics
Luleå University of Technology
Luleå SE-97187, Sweden

Y. Wang
Key Laboratory of Science and Technology on Wear and Protection of Materials
Lanzhou Institute of Chemical Physics
Chinese Academy of Sciences
Lanzhou 730000, China

J. Zhang
Center of Materials Science and Optoelectronics Engineering
University of Chinese Academy of Sciences
Beijing 100049, China

Table of contents

Figure S1. TEM image of GMS-assembled coating.
Figure S2. Fabrication and structure analysis of GMS-assembled coating.
Figure S3. Strain analysis of GMS-assembled coating.
Figure S4. Structural characterizations of GMS-assembled coating from other coated ball position.
Figure S5. Structural characterizations of GMSs from another position in other ball.
Figure S6. Structures and properties of DLC0, DLC12 and DLC25.
Figure S7 Friction behaviors for DLC samples against GMS-assembled coating.
Figure S8. Friction coefficient curves for GMS-assembled coating against DLC25 under different conditions.
Figure S9. TEM cross-section characterization of GMS-assembled coating against DLC25 after friction.
Figure S10. 2D morphologies of DLC surfaces.
Figure S11. Structural characterization of GMS-assembled coating against DLC25 before and after friction.
Figure S12 Sliding simulation of a polycrystalline graphene layer with different face orientations on DLC.
Figure S13. DFT calculations of bilayer graphene and H-diamond interfaces.
Figure S14. PES corrugation of 0° and 30.0° bilayer graphene.
Figure S15. MD simulation of GMS-assembled flake sliding on DLC.
Figure S16. MD simulation of CBG-assembled flake sliding on DLC.
Text S1: GMS formation on a sphere.
Text S2: MD simulations of GMS-assembled flake sliding on DLC.
Text S3: DFT calculations of GMS face and DLC25 interface.
References

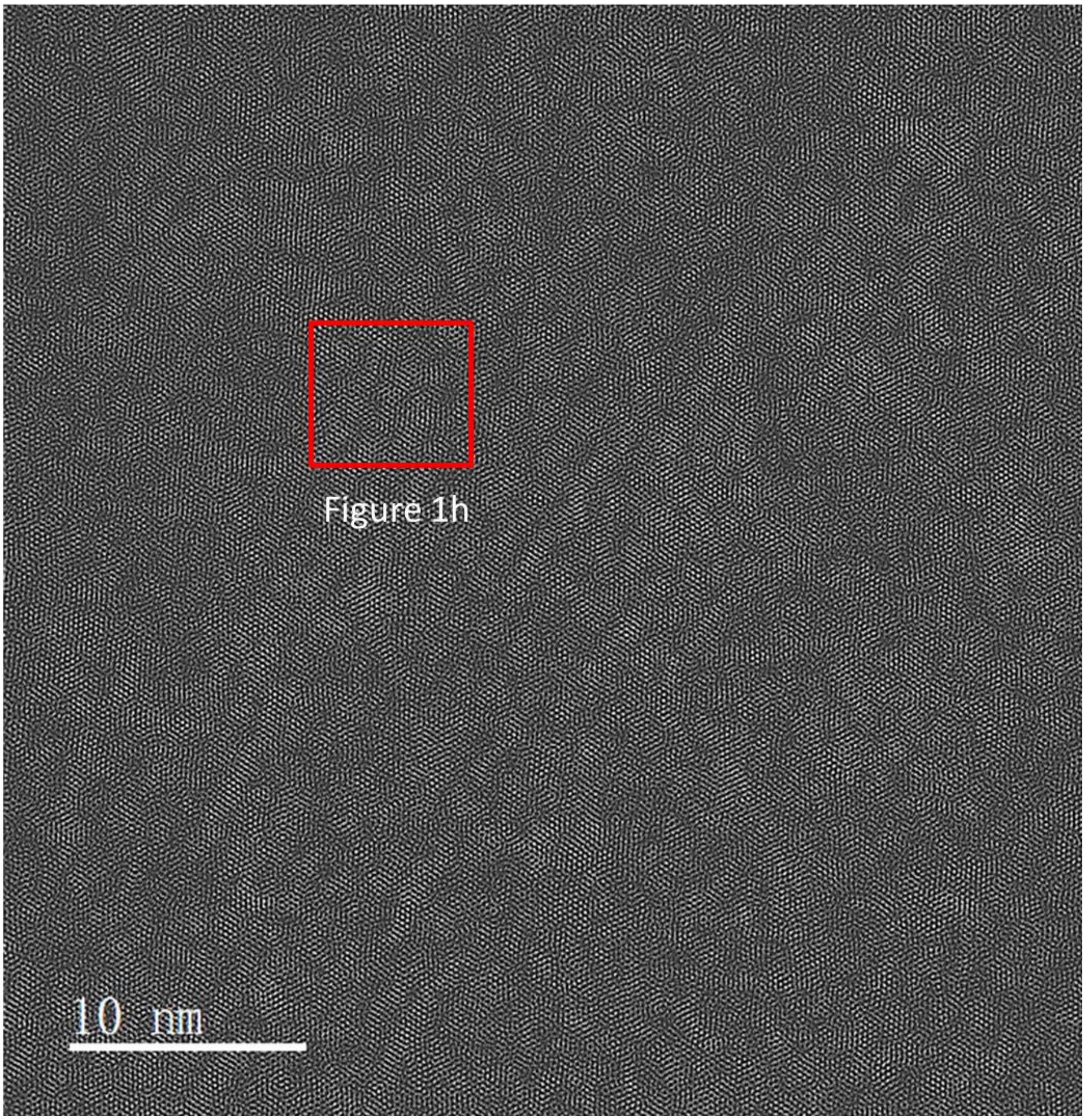

Figure S1. TEM image of GMS-assembled coating. The Figure 1h is cut from the Supplementary Figure 1 and the FFT results of Figure 1j is obtained at large area of 45 nm×45 nm (i.e. 2025 nm^2).

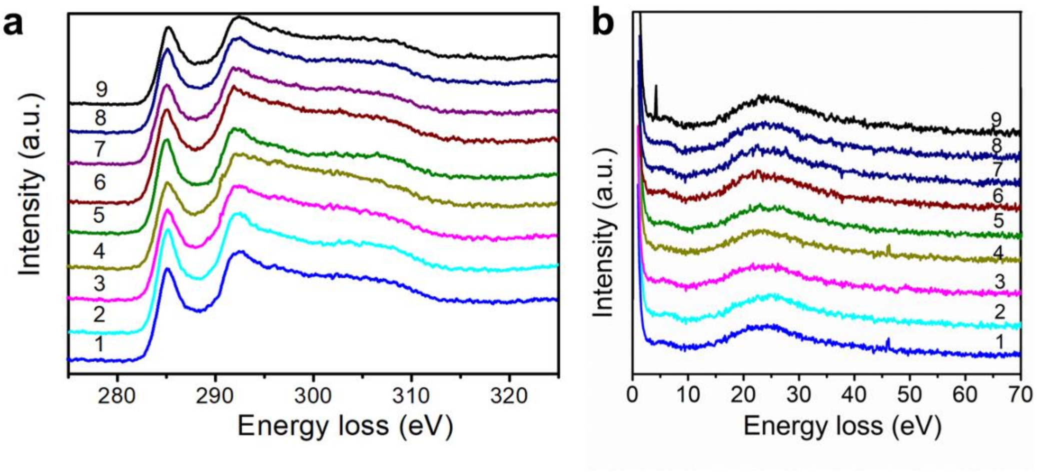

Figure S2. Fabrication and structure analysis of GMS-assembled coating. a,b) Recorded low-loss and zero-loss EELS spectra of GMS coating as marked in Figure 1. The carbon K-edge spectrum shows signals of graphene with a peak in the range of 285-290 eV and a other peak at 292 eV attributable to the 1s-π^* and the 1s-σ^* electronic transitions.[1]

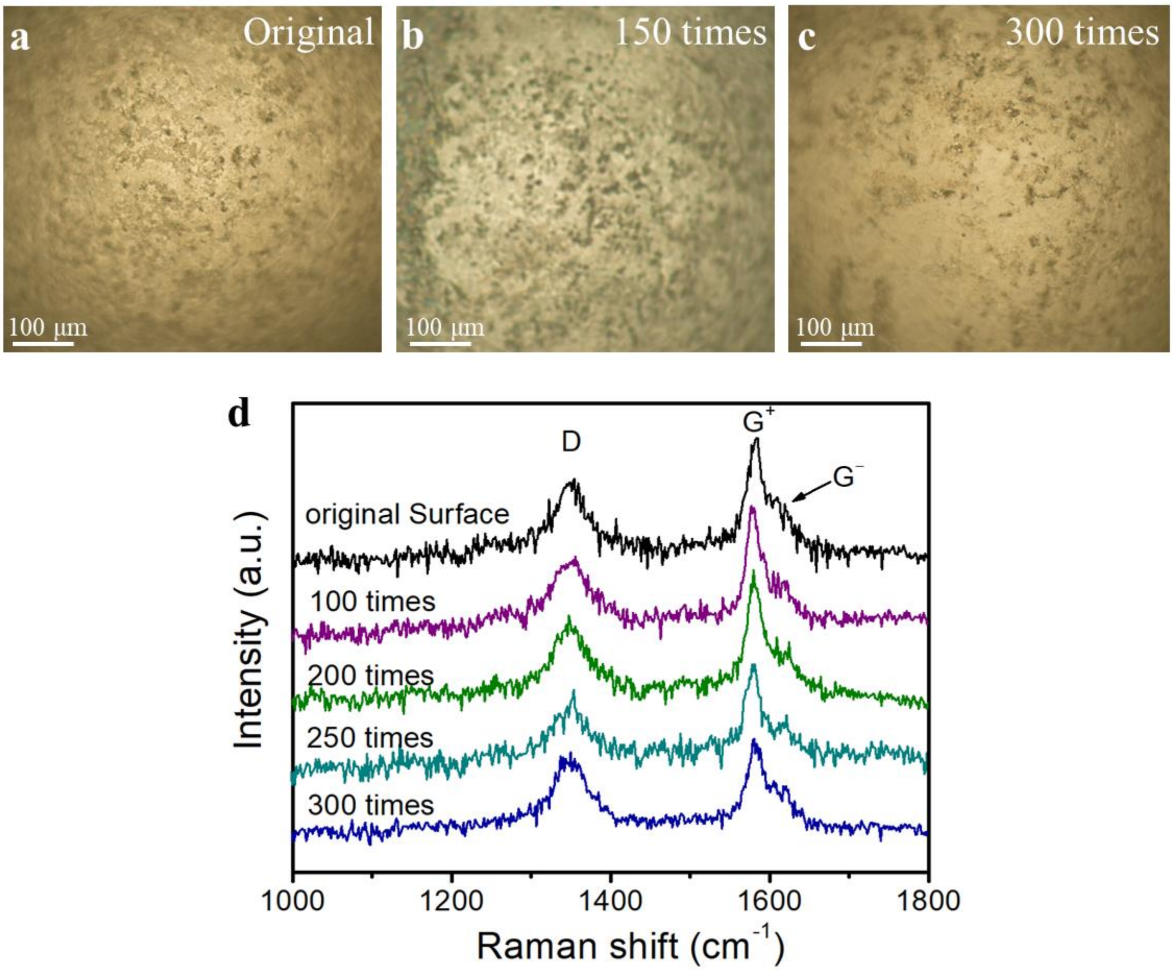

Figure S3. Strain analysis of GMS-assembled coating. a-c) original surface, and surfaces after 150 and 300 times taping. d) Raman spectra of newly formed ball surface when original ball surface being gradually glued for 300 times. The splitting of Raman G peak into two subbands G^+ and G^- is analogous to that induced by curvature in nanotubes.[2,3] These subbands shift with increasing strain and their splitting increases.[2,3]

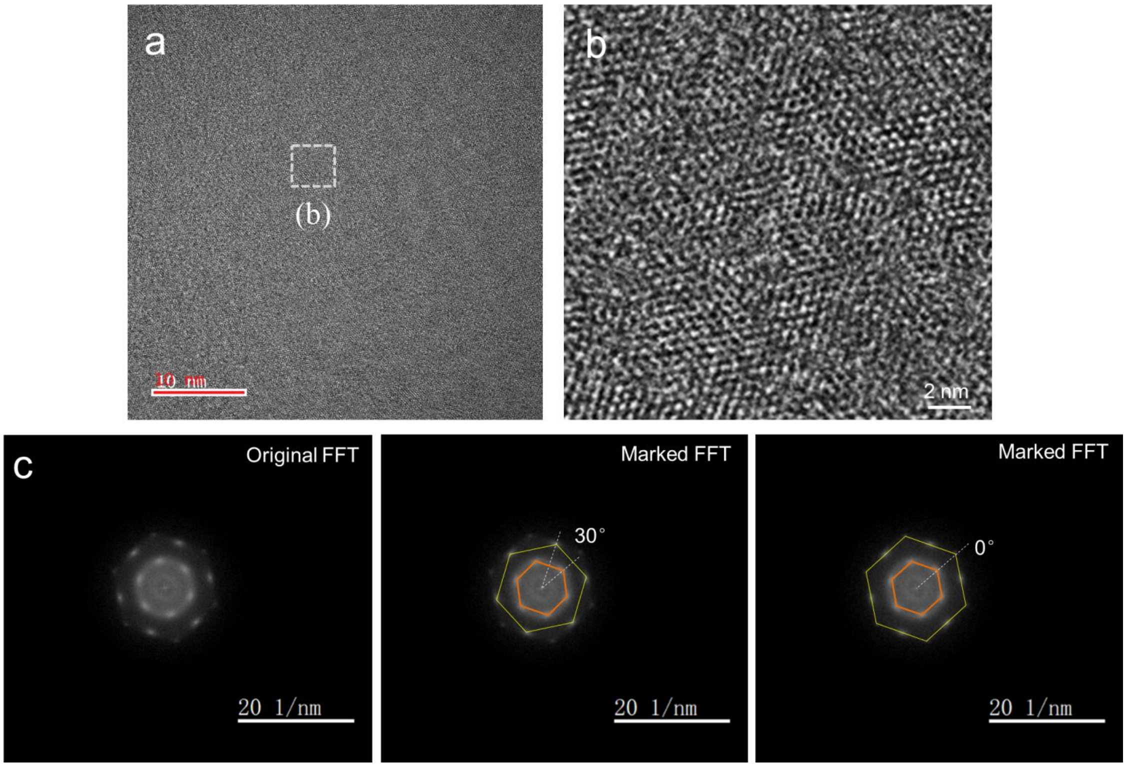

Figure S4. Structural characterizations of GMS-assembled coating from other coated ball position. a) TEM images. b) is marked in (a). c) Original and marked FFT results of (a).

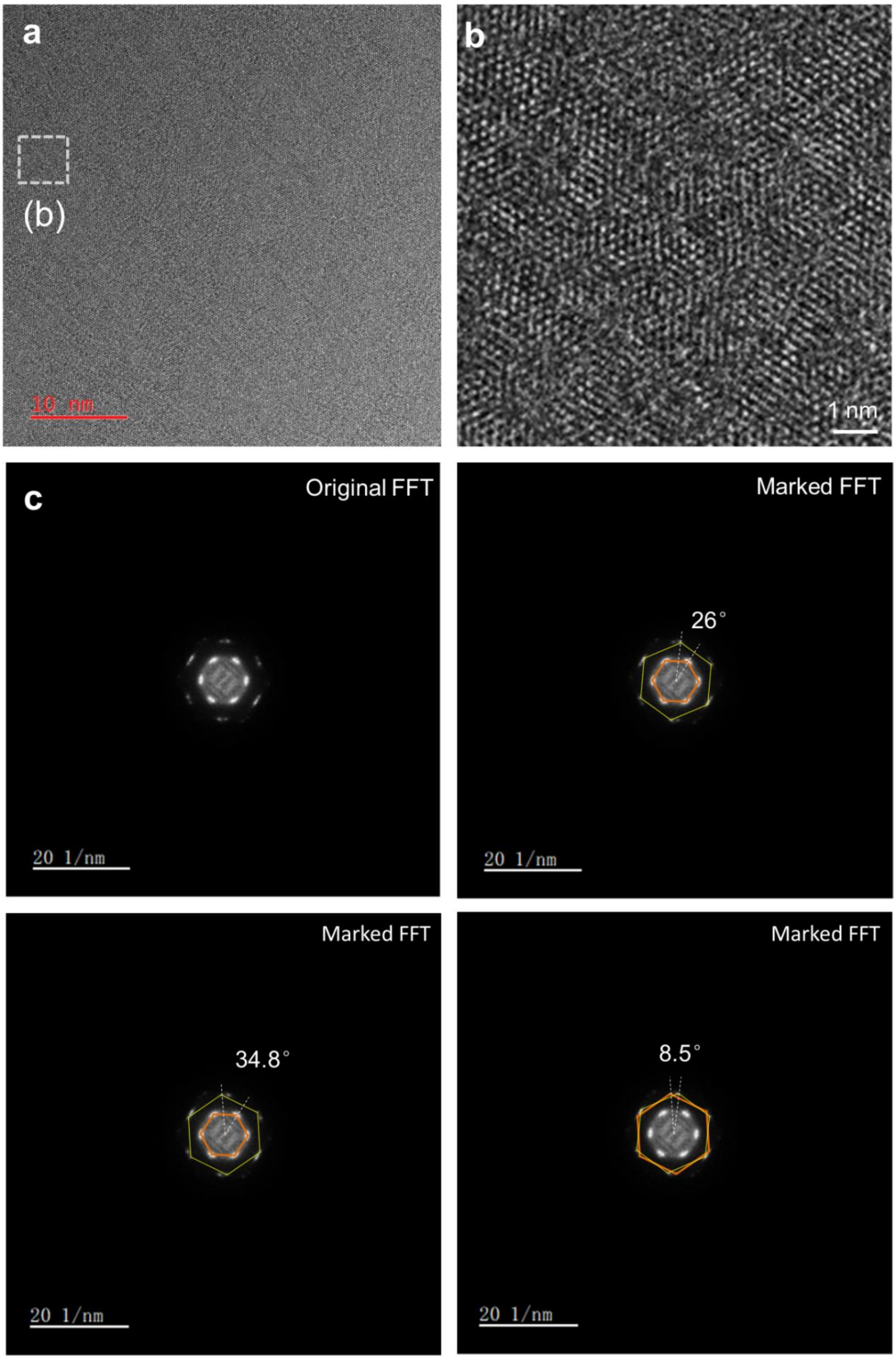

Figure S5. Structural characterizations of GMSs from another position in other ball. a) TEM images. b) is marked in (a). c) Original and marked FFT results of (a).

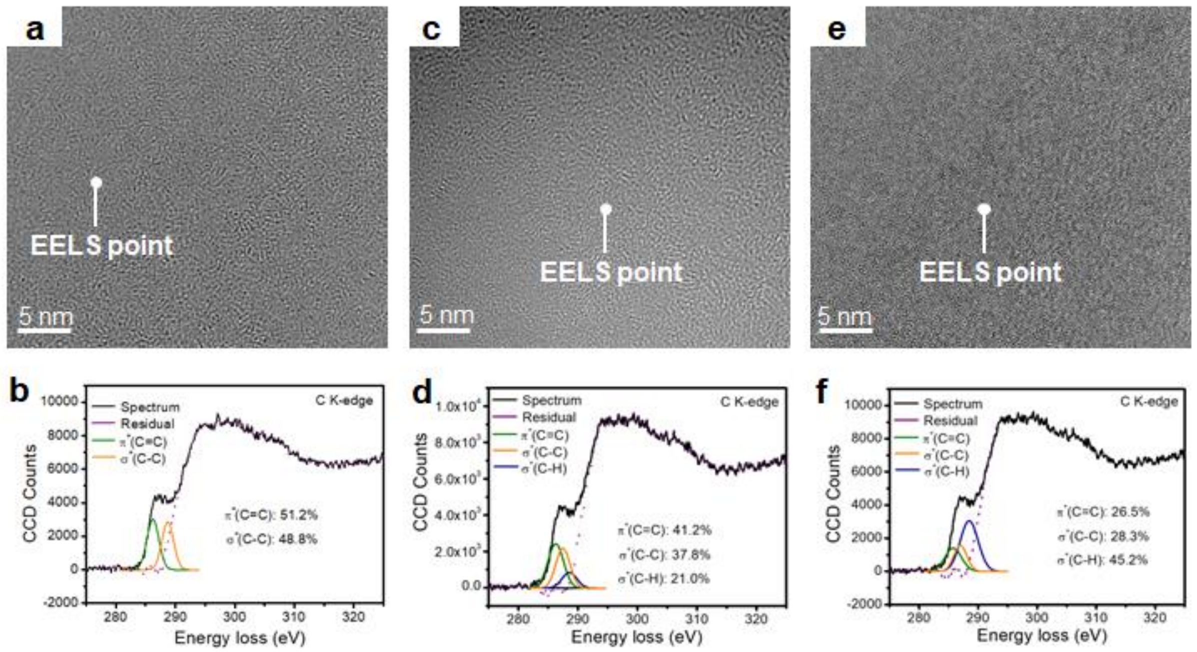

Figure S6. Structures and properties of DLC0, DLC12 and DLC25. a, c, e) Cross-section TEM images of DLC0, DLC12 and DLC25. b, d, f) Core-loss EELS spectrum and fitting results of DLC0, DLC12 and DLC25. The C-K edges are fitted by three Gaussian peaks C=C at 285.5 eV, C-H at 287 eV, and C-C at 292.5 eV.

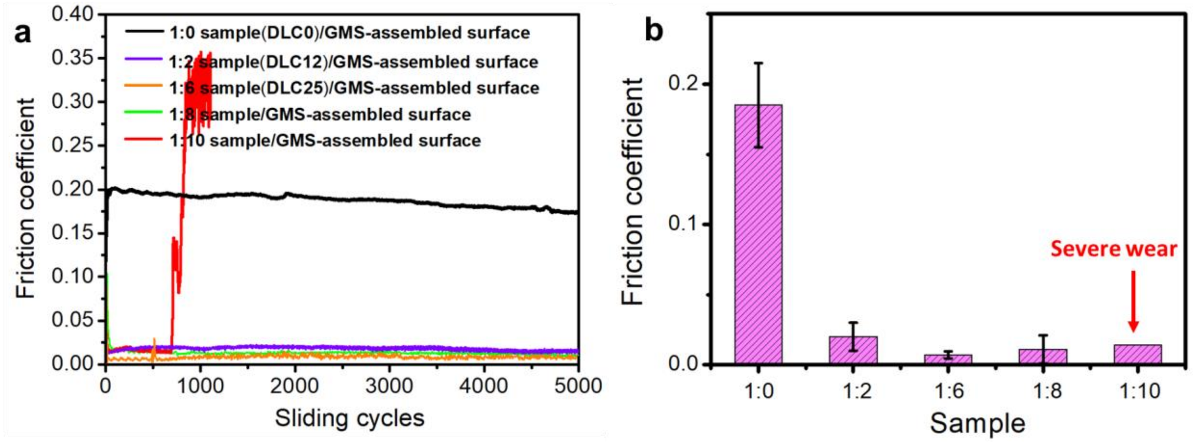

Figure S7 Friction behaviors for DLC samples against GMS-assembled coating. (a) Friction coefficient curves. (b) Average friction coefficients of (a).

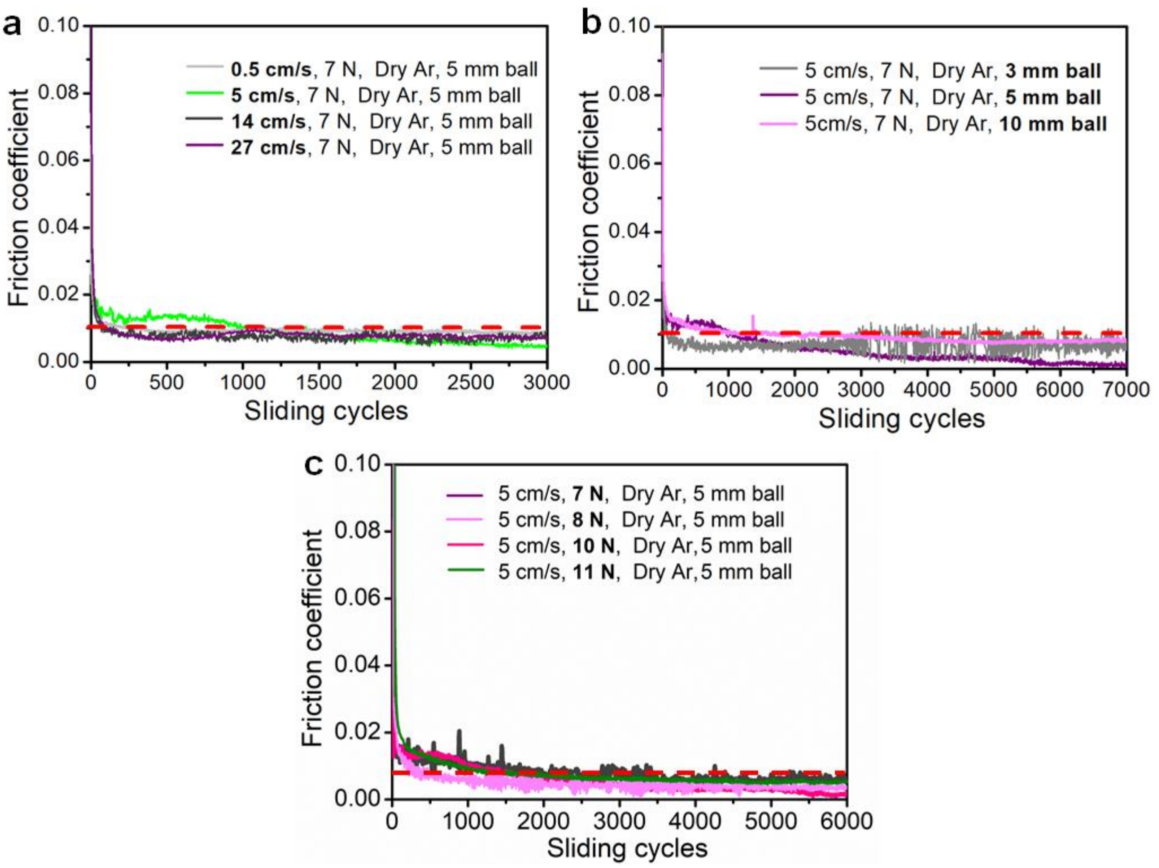

Figure S8. Friction coefficient curves for GMS-assembled coating against DLC25 under different conditions. a) Sliding velocity. b) Different contact area obtained by varying the ball diameter. c) External load.

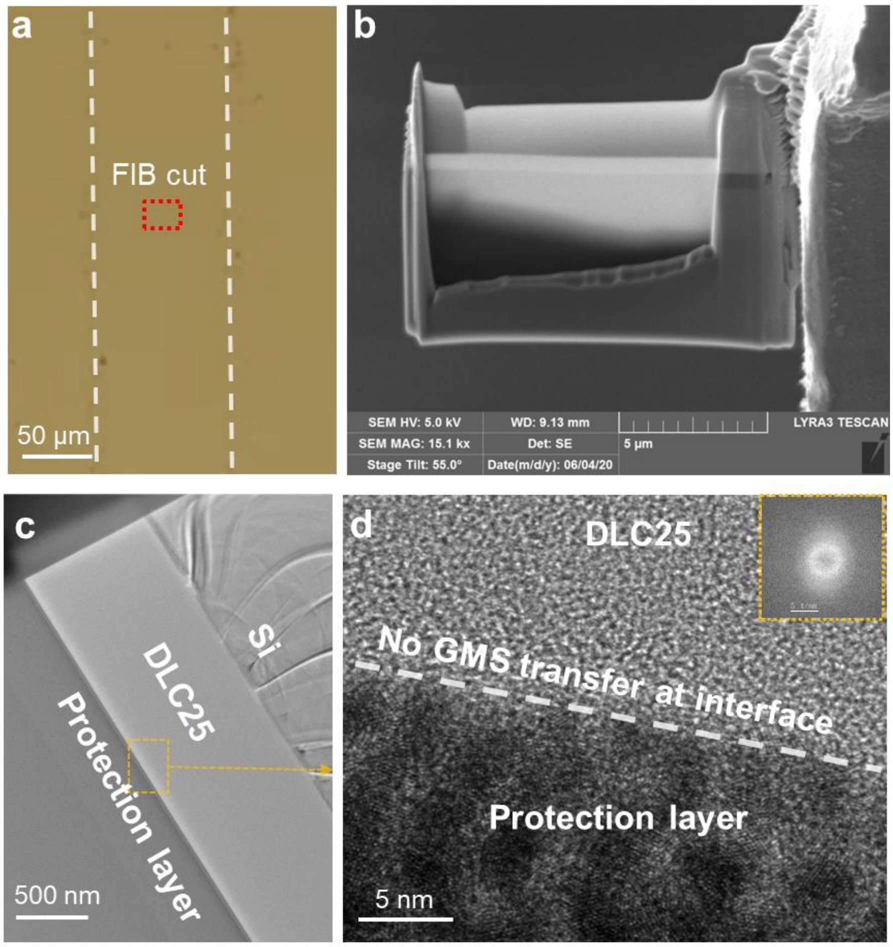

Figure S9. TEM cross-section characterization of GMS-assembled coating against DLC25 after friction. a) Wear track under the condition of 7 N, 5 cm/s and 5 mm ball. b) FIB-SEM images marked in (a). c, d) corresponding TEM results.

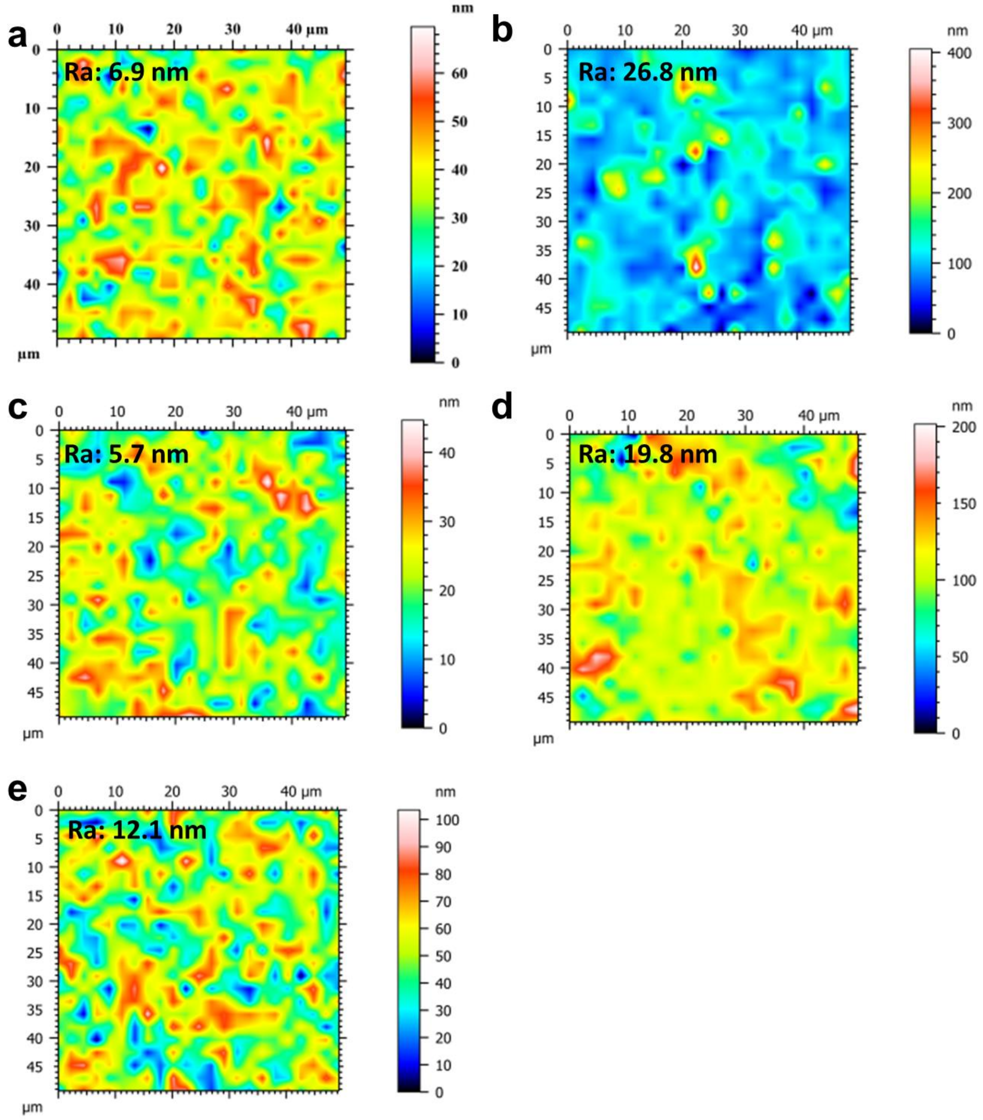

Figure S10. 2D morphologies of DLC surfaces. a, b) 2D morphologies of DLC0 surface and frictional surface in GMS-assembled coating/DLC0 tribo-pairs. c, d) 2D morphologies of DLC12 surface and frictional surface in GMS-assembled coating/DLC12 tribo-pairs. e) 2D morphologies of DLC12 surface in ZrO_2/DLC25 surfaces.

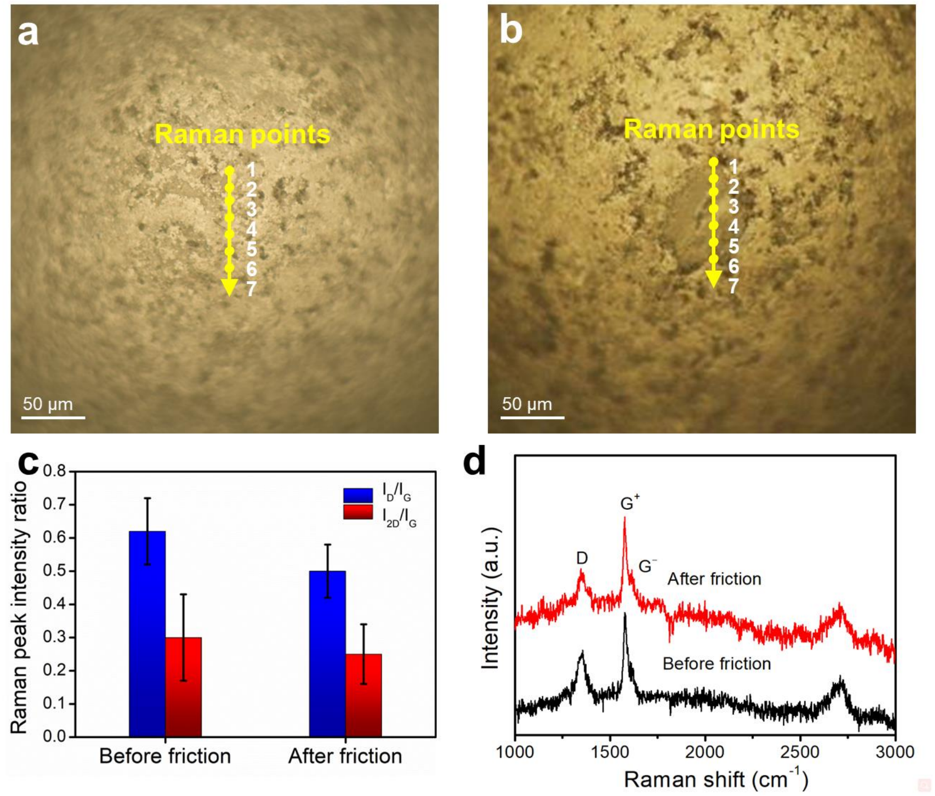

Figure S11. Structural characterization of GMS-assembled coating against DLC25 before and after friction. a, b) Raman measured positions before and after friction under the condition of 7 N, 5 cm/s and 5 mm ball. c, d) Raman peak intensity ratios and typical Raman spectra corresponding to (a, b), respectively.

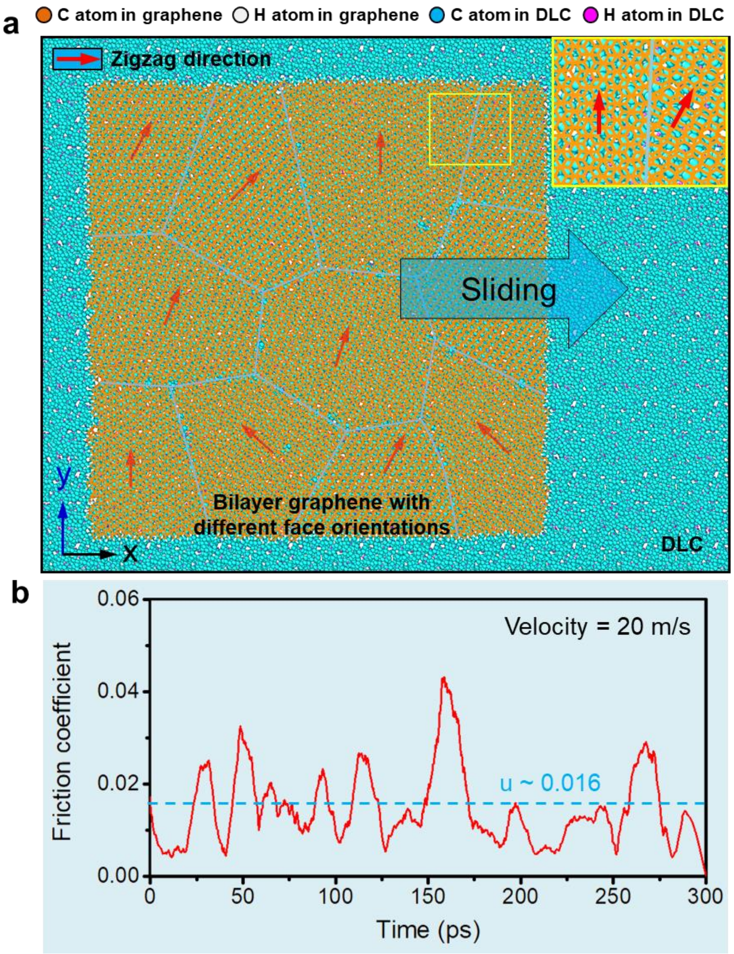

Figure S12. Sliding simulation of a polycrystalline graphene layer with different face orientations on DLC. (a) Simulation model DLC. (b) Friction results.

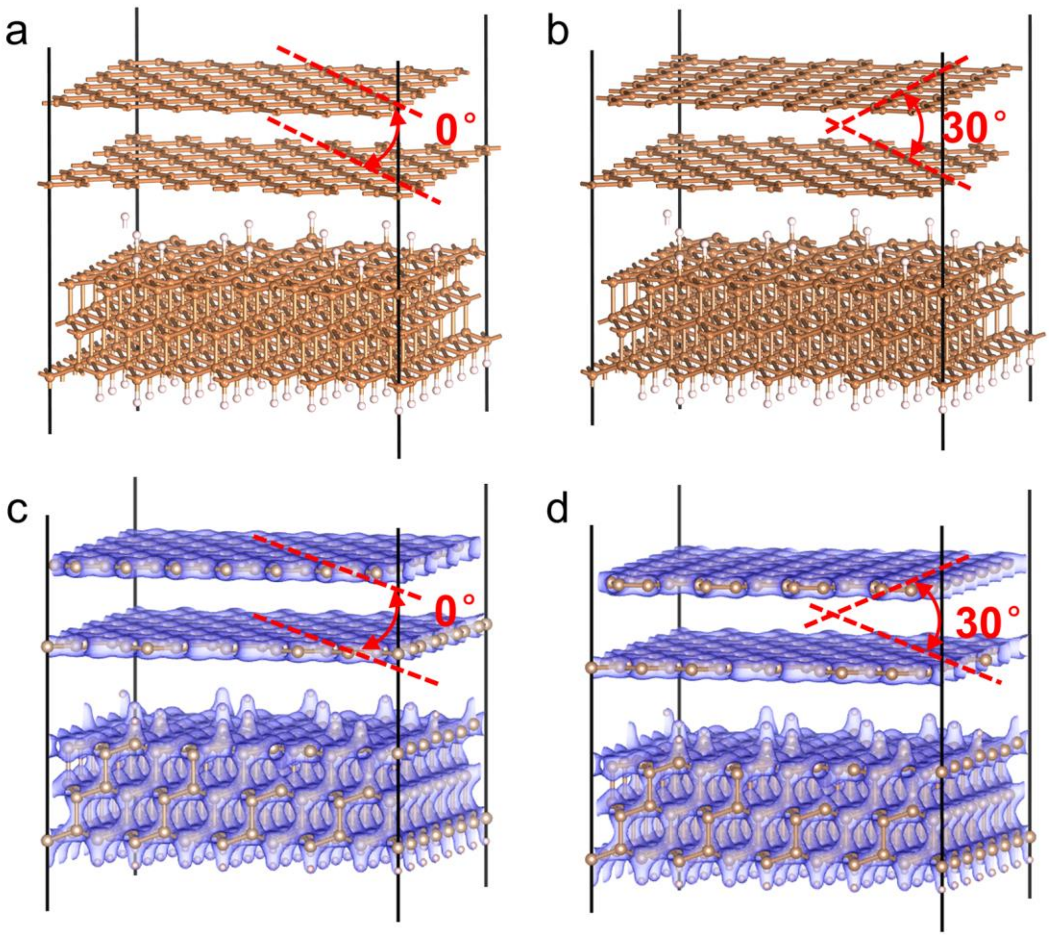

Figure S13. DFT calculations of bilayer graphene and H-diamond interfaces. a, b) Models of 0° and 30.0° bilayer graphene on H-diamond (H: 25.at.%). c,d) 3D electron density images of (a) and (b), respectively.

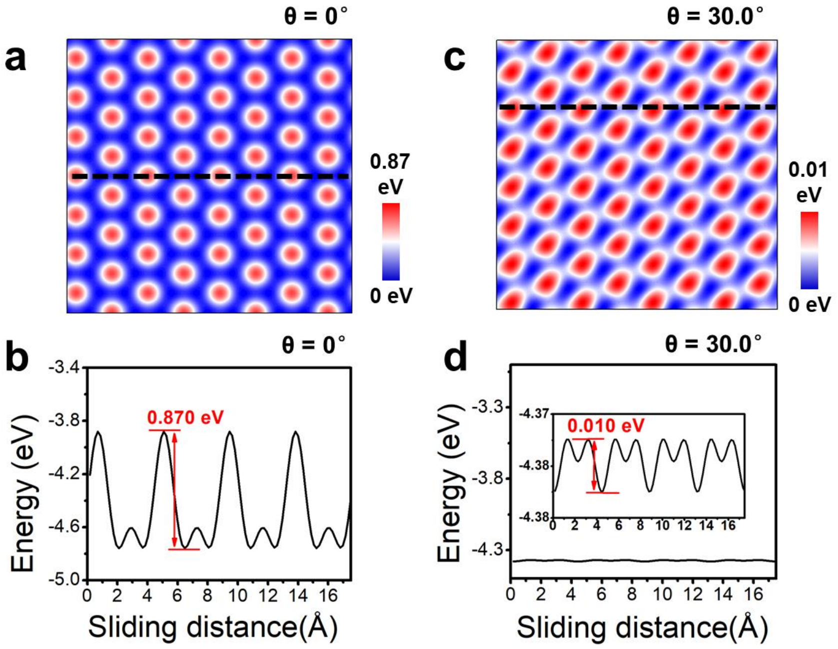

Figure S14. PES corrugation of 0° and 30.0° bilayer graphene. a, b) PES corrugation their PES corrugation curves of 0° bilayer graphene. c,d) PES corrugation their PES corrugation curves of 30.0° bilayer graphene.

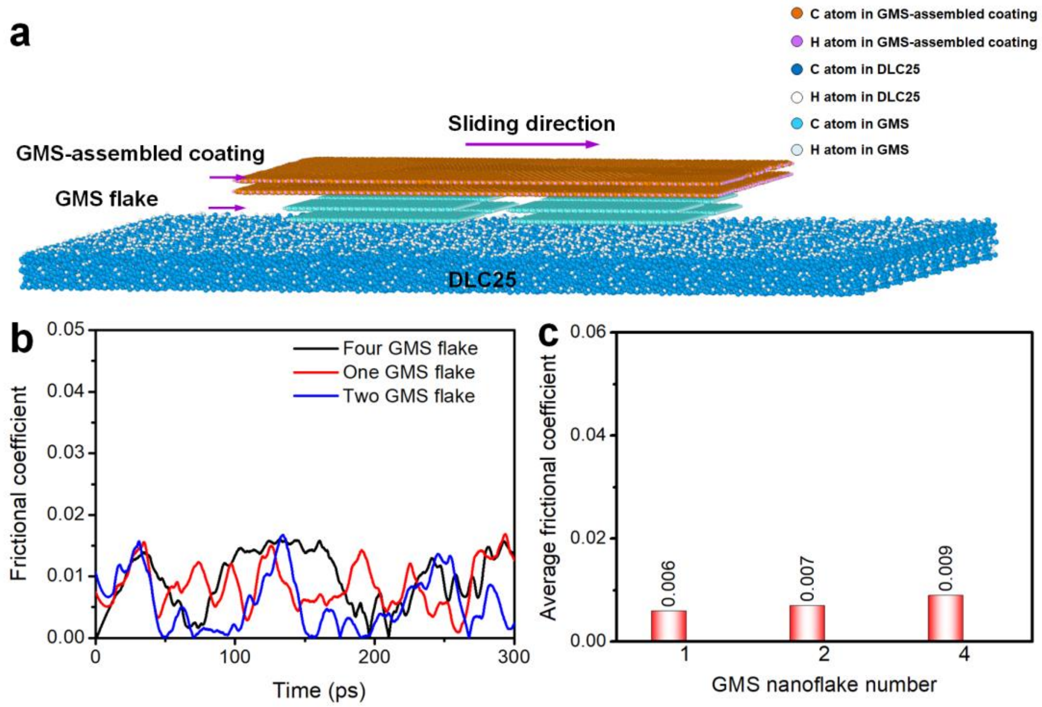

Figure S15. MD simulation of GMS-assembled flake sliding on DLC. a) Friction models, b) Friction results. c) Average friction results.

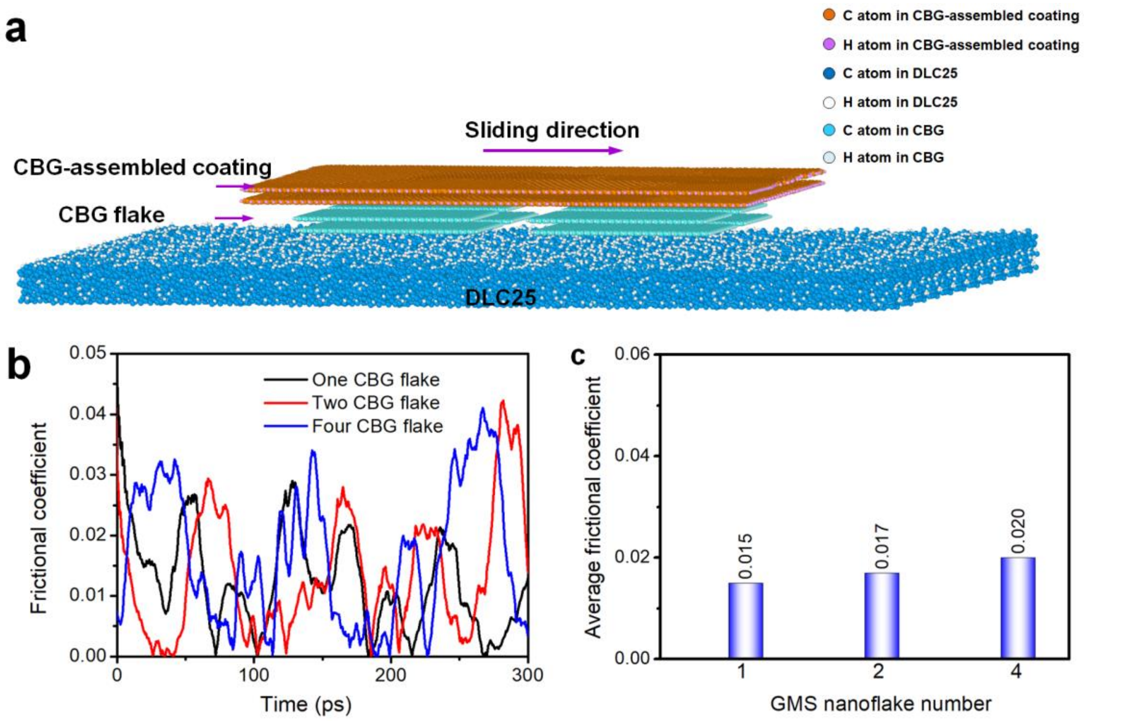

Figure S16. MD simulation of CBG-assembled flake sliding on DLC. a) Friction models, b) Friction results. c) Average friction results.

Text S1: GMS formation on a sphere.

In order to reveal the formation mechanism of GMSs on a sphere, we investigated the structural evolution of circular bilayer graphene (~40 Å of radius) on a rigid hemisphere using MD simulations (Figure 5). The adaptive interatomic reactive empirical bond-order (AIREBO) potential[4] was used for graphene, rigid hemisphere, while the Lennard-Jones (L-J) potentials expressed by $U(r) = 4\varepsilon\{(\sigma/r)^{12} - (\sigma/r)^{6}\}$ were applied to describe the interactions between them. For the parameters of L-J potential, $\sigma_{C\text{-}C}$ = 3.4 Å, $\varepsilon_{C\text{-}C}$ = 2.84 meV. The simulation under a NVT ensemble with the constant temperature of 300 K controlled by Nosé-Hoover thermostat was performed. The relative angle between bilayer graphene, the strain for every atom in bilayer graphene and the total energy during the simulation process were analyzed to estimate the change of bilayer graphene. All simulations were carried out in LAMMPS software.[5]

Text S2: MD simulations of GMS-assembled flake sliding on DLC.

We investigated the sliding behaviors of eight nano-sized GMS-assembled flake and eight nano-sized CBG-assembled flake on H-DLC substrates with H: 0 at.%, 12 at.% and 25 at.% (DLC0, DLC12 and DLC25) (Figure 6). For GMS-assembled and CBG-assembled flakes, we constructed them based on incommensurate and commensurate bilayer graphene flakes, respectively (Figure 6a-c), which was consistent with the experimental data. The H-DLC substrates with different hydrogen content were obtained by annealing from 4500 K to 300 K. In the sliding model, the H-DLC substrate was composed of ~100000 atoms with a dimension of 256 Å × 256 Å along the x and y directions, which contained two segments: the constrained bottom layer with the height of ~2 Å and the free upper layer of ~8 Å. And, the GMS-assembled or CBG-assembled flake with the length of ~150 Å was driven to slide against the substrate along x direction with the constant velocity of 20 m/s. Periodic boundary condition was employed in the x and y directions. For the sliding simulations, the AIREBO and L-J potentials were used to describe the atomic bond interactions and the inter-layer van der Waals interactions, respectively. For L-J potential, $\sigma_{C\text{-}C}$ = 3.4 Å, $\varepsilon_{C\text{-}C}$ = 2.84 meV, $\sigma_{C\text{-}H}$ = 3.025 Å and $\varepsilon_{C\text{-}H}$ = 1.376 meV. In the sliding simulations under a NVT ensemble, the system temperature was maintained at 300 K using Nosé-Hoover thermostat.[6,7] The forces along the x and z directions for graphene flakes were recorded as the friction and normal forces. Additionaly, we also investigated the effect of the different amount of bilayer graphene flakes on sliding behavior (Figures S13-14, Supporting Information).

Text S3: DFT calculations of GMS face and DLC25 interface.

Bilayer graphene/H-diamond (H~25 at.%) model contained about 600 atoms and possessed the size of 17.3490 Å × 17.3468 Å (Figure 7 and Figure S11, Supporting Information). To avoid interactions between the sheet and its periodic images, the vacuum of more than 15 Å for all models were added along the *z*-direction. For bilayer graphene/H-diamond systems, we calculated their interaction energies using the formula of $\Delta E = E_{AB}-(E_A+E_B)$, in which E_{AB}, E_A and E_B were the total energies of bilayer graphene/H-diamond systems and the corresponding components. To evaluate the influence of 0° and 30° bilayer graphene, we simulated electron density and their difference relative to single components, and scaned the interaction potential energy surface (PES) corrugation for bilayer graphene and bilayer graphene/ H-diamond systems. For bilayer graphene, we also considered the effect of different twisted angles (0° and 30°) on electron density distribution. Easily analyze the distinction between them, we obtained the cross profiles of electron density difference maps along different z positions (z = 0.50, 0.48, 0.46, 0.44) in the x-y plane. To obtain accurate PES results under moderate calculation costs, we firstly scanned the PES using MD simulations, and then calculated the accurate energies at maximum and minimum using DFT method and finally draw PES maps. All the density functional theory (DFT) calculations were performed in the Vienna ab intio simulation package (VASP).[8,9] The generalized gradient approximation (GGA) with Perdew-Burke-Ernzerhof (PBE) functional[10] was used to describe the exchange-correlation contributions. To obtain structural optimizations, electron density distribution calculations and PES scan, the energy cutoff 400 eV for the plane-wave basis was selected, and the energy and force thresholds were set to 10^{-4} eV and 0.05 eV/Å.

References

[1] D. G. McCulloch, J. L. Peng, D. R. Mckenzie, S. P. Lau, D. Sheeja, B. K. Tay, *Phys. Rev. B* **2004**, *70*, 085406.

[2] A. C. Ferrari, D. M. Basko, *Nat. Nanotechnol.* **2013**, *8*, 235.

[3] T. M. G. Mohiuddin, A. Lombardo, R. R. Nair, A. Bonetti, G. Savini, R. Jalil, N. Bonini, D. M. Basko, C. Galiotis, N. Marzari, K. S. Novoselov, A. K. Geim, A. C. Ferrari, *Phys. Rev. B* **2009**, *79*, 205433.

[4] S. J. Stuart, A. B. Tutein, J. A. Harrison, *J. Chem. Phys.* **2000**, *112*, 6472.

[5] S. Plimpton, *J. Comput. Phys.* **1995**, *117*, 1.

[6] A. Nosé, S. *J. Chem. Phys.* **1984**, *81*, 511.

[7] W. G. Hoover, *Phys. Rev. A* **1985**, *31*, 1695.

[8] G. Kresse, J. Furthmüller, *Phys. Rev. B* **1996**, *54*, 11169.

[9] G. Kresse, J. Hafner, *Phys. Rev. B* **1993**, *47*, 558.

[10] J. P. Perdew, K. Burke, M. Ernzerhof, *Phys. Rev. Lett.* **1996**, *77*, 3865.
